# High-Sensitivity Refractive Index Sensor with Dual-Channel Based on Surface Plasmon Resonance Photonic Crystal Fiber

**DOI:** 10.3390/s24155050

**Published:** 2024-08-04

**Authors:** Fengmin Wang, Yong Wei, Yanhong Han

**Affiliations:** 1 Liren College, Yanshan University, Qinhuangdao 066004, China; hanyanhong@ysu.edu.cn; 2 School of Information Science and Engineering, Yanshan University, Qinhuangdao 066004, China; wp921@ysu.edu.cn

**Keywords:** photonic crystal fiber, surface plasmon resonance, wavelength sensitivity

## Abstract

In order to achieve a high-precision synchronous detection of two different refractive index (RI) analytes, a D-type surface plasmon resonance (SPR) photonic crystal fiber (PCF) RI sensor based on two channels is designed in this paper. The sensor uses a D-shaped planar region of the PCF and a large circular air hole below the core as the sensing channels. Surface plasmon resonance is induced by applying a coating of gold film on the surface. The full-vector finite-element method (FEM) is used to optimize the structural parameters of the optical fiber, and the sensing characteristics are studied, including wavelength sensitivity, RI resolution, full width at half maximum (*FWHM*), figure of merit (*FOM*), and signal-to-noise ratio (*SNR*). The results show that the channel 1 (Ch 1) can achieve RI detection of 1.36–1.39 in the wavelength range of 1500–2600 nm, and the channel 2 (Ch 2) can achieve RI detection of 1.46–1.57 in the wavelength range of 2100–3000 nm. The two sensing channels can detect independently or simultaneously measure two analytes with different RIs. The maximum wavelength sensitivity of the sensor can reach 30,000 nm/RIU in Channel 1 and 9900 nm/RIU in Channel 2. The RI resolutions of the two channels are 3.54 × 10^−6^ RIU and 10.88 × 10^−6^ RIU, respectively. Therefore, the sensor realizes dual-channel high- and low-RI synchronous detection in the ultra-long wavelength band from near-infrared to mid-infrared and achieves an ultra-wide RI detection range and ultra-high wavelength sensitivity. The sensor has a wide application prospect in the fields of chemical detection, biomedical sensing, and water environment monitoring.

## 1. Introduction

Photonic crystal fiber (PCF) [1] has a periodic array of pores, which are closely arranged in the two-dimensional direction and maintain the structure along the axis, so it is also called microstructured optical fiber (MOF) [2]. Since its inception, PCF has received great attention from the majority of researchers. The new characteristics of low-optical loss and high-optical nonlinearity make PCF more widely used in sensing, detection, optical fiber communication, nonlinear optics, and many other fields [3,4,5,6]. Surface plasmon resonance (SPR) is a physical phenomenon that occurs when the wave vector of incident light matches the wave vector of the surface plasmon wave. The wave vector of surface plasmon waves depends on the refractive index (RI) of the metal and the material combined with the metal so that SPR technology can monitor the change in RI caused by the combination of the material with the metal surface. With the discovery of SPR, the sensor based on surface plasmon resonance photonic crystal fiber (SPR-PCF) came into being. Because of its small size, strong anti-electromagnetic interference ability, high sensitivity, fast response, and other advantages, it has been widely used in the field of sensing [7]. Moreover, the SPR-PCF sensors can be flexibly designed. By changing the design parameters of PCF, the phase matching of the light wave and surface plasma wave can be adjusted so that the RI detection range and sensing band of the sensor can be changed and the sensing performance can be improved [8,9].

With the rise of SPR-PCF sensors, more and more researchers are engaged in this field. In recent years, SPR-PCF sensors have entered an unprecedented stage of development. Researchers from various countries have conducted in-depth research in many sensing fields, such as temperature [10], biology [11], gas [12], pressure [13], and so on [14,15,16], and they have achieved a large number of scientific research results [17]. In 2017, D.F. Santos et al. [18] designed a sensor consisting of a D-type SPR-PCF with metamaterial layers. They demonstrated that the loss and operation wavelength could be controlled in a wide range by changing the relative proportion of different materials that comprise the metamaterial. In 2018, Yundong Liu et al. [19] proposed a gold-plated D-shaped PCF RI sensor with a circular layout of pores and studied the sensing characteristics of the sensor using FEM. In 2019, Shun Wang et al. [20] proposed and analyzed a SPR sensor based on symmetrical side-polished dual-core PCF. In 2022, Ahmed A. Saleh Falah et al. [21] proposed and comprehensively investigated a high-sensitivity gold-coated eccentric core D-shaped SPR-PCF sensor based on uniform circular glass capillaries and solid rods, which had high linearity.

With the diversification of detection target samples and the increasing demand for detection, traditional single-channel SPR-PCF RI sensors cannot meet the needs of practical detection. Therefore, multi-channel SPR-PCF RI sensors have emerged. In 2020, Pibin Bing et al. [22] proposed a double-sample synchronous detection sensor based on up-core PCF, which could simultaneously detect double samples with an RI of 1.34–1.39 in the wavelength range from 550 nm to 900 nm. The maximum wavelength sensitivity could reach 8300 nm/RIU. In 2022, Hairui Fang et al. [23] designed a symmetrical dual-channel D-type PCF-SPR sensor that could simultaneously detect the analyte RI in two non-interference channels. This sensor could achieve an RI detection of 1.33–1.40 in the wavelength range of 500 nm to 1100 nm, with a maximum wavelength sensitivity of 14,000 nm/RIU. In 2023, Farhan Mumtaz et al. [24] proposed a windmill-shaped three-channel SPR sensor for simultaneous detection. The RI detection ranges of the channels were 1.30–1.34, 1.35–1.39, and 1.40–1.44, respectively. In the near-infrared region, the maximum wavelength sensitivities of the three channels were 3292 nm/RIU, 6664 nm/RIU, and 10,243 nm/RIU, respectively. In 2024, Mohd Fahmi Azman et al. [25] developed a dual-channel single-polarization PCF-SPR sensor. The maximum wavelength sensitivity of the sensor in two channels was 11,000 nm/RIU. In the 550–1100 nm band, the detection range of analyte RI was 1.33–1.41. However, the performance of these sensors is not remarkable. The two channels have the same RI detection range, and most of their resonance wavelengths are in the visible and near-infrared bands. Therefore, a sensor with high-wavelength sensitivity, wide RI detection range, and the ability to work in a wider wavelength band is needed.

In this paper, we propose a high-sensitivity, dual-channel, D-shape PCF-SPR RI sensor that can work in the ultra-wide wavelength range from near-infrared to mid-infrared. A large circular pore located in the middle of the fourth layer pores serves as a sensing channel. Another sensing channel is the polished D-shaped plane area. The two sensing channels can independently detect high- and low-RI analytes, or simultaneously detect two analytes with different RIs. The full-vector FEM is used to optimize the structure parameters of the sensor. The sensing characteristics of the optimized sensor are numerically simulated. The wavelength sensitivity, RI resolution, *FWHM*, *FOM*, and *SNR* of the sensor are calculated. The calculation results show that the sensor has an excellent sensing performance. The sensor has a maximum wavelength sensitivity of up to 30,000 nm/RIU in Channel 1 and 9900 nm/RIU in Channel 2, as well as excellent linearity. In addition, the sensor can achieve an ultra-wide RI detection range in the ultra-long wavelength range of 1500–3000 nm, with the RI detection range of 1.36–1.39 in Channel 1 and 1.46–1.57 in Channel 2.

## 2. Structure Design and Theoretical Modeling

**Figure 1:**
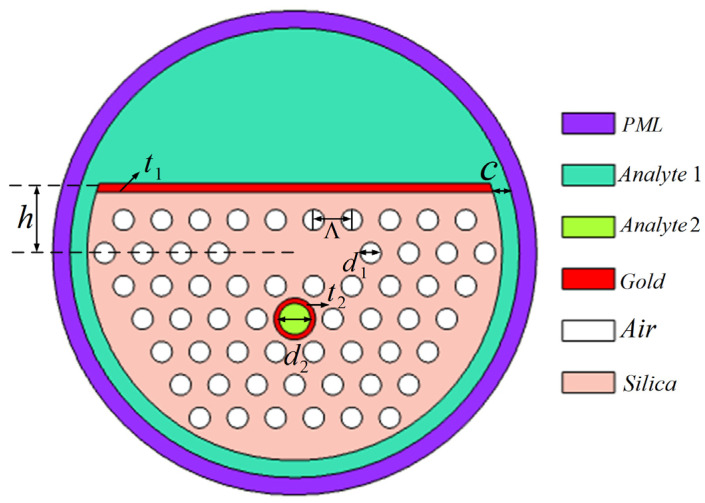
The cross-sections of the proposed SPR-PCF dual-channel refractive index sensor.

The cross-section of the D-type SPR dual-channel sensor based on PCF designed in this paper is shown in Figure 1. The PCF structure consists of five layers of circular air holes arranged in hexagons. The diameter of the air hole is *d_1_*. The lattice constant is Ʌ. The polished D-shaped structure on the upper side of the optical fiber is located *h* away from the core; that is, the polishing depth is *h*. This D-type PCF-SPR sensor has two sensing channels. Channel 1 is located in the D-shaped plane area outside the optical fiber, and the thickness of the sensing layer is c. In order to excite surface plasmons, a gold layer with a thickness of *t_1_* is coated on the D-shaped plane. Channel 2 is a large circular air hole with a diameter of *d_2_*, which is located in the middle of the fourth layer air hole on the lower side of the core. The outer side of Channel 2 is coated with a layer of gold nanofilm with a thickness of *t_2_* to promote the SPR effect. The RI of the analyte in Channel 1 is *n_1_*, and the RI of the analyte in Channel 2 is *n_2_*. In comparison to other geometric structures, the D-type PCF discussed in this paper is designed with common circular air holes and a symmetrical layout, making it simpler to manufacture. However, the metal plasma material in the D-shaped region is prone to oxidation when exposed to air, potentially affecting the sensor’s stability. To address this issue, gold, known for its stable chemical properties, has been chosen as the plasma material.

**Figure 2:**
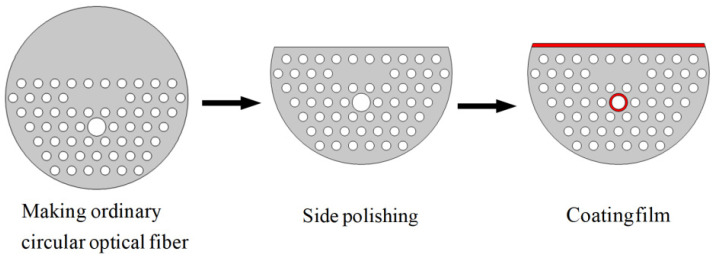
Schematic for the fiber-fabrication process. The white circle represents the air holes. The gold layer is marked in red.

At present, the manufacturing technology of PCF and the metal-coating method are relatively mature, and the production process of the PCF-SPR sensor proposed in this paper can be divided into three steps. One is to draw a conventional circular PCF. There are many techniques for fabricating a conventional circular PCF, such as the stack-and-draw method [26], the sol–gel method [27], drilling [28], and extrusion [29]. Among them, the stack-and-draw technique has become the most important and commonly used method for producing PCF due to its simple operation, low cost, and strong flexibility. The sol–gel method proposed by El Hamzaoui et al. in 2012 can manufacture any PCF structure and can freely adjust the spacing, size, and shape of air holes. Therefore, the PCF and its air-hole structure can be fabricated by the stack-and-draw method or sol–gel method. Next, the desired D-type PCF can be fabricated by the side wheel grinding and polishing technology. The last step is to coat the two sensing channels with a layer of gold film. In previous studies [30,31,32,33], the technology of selectively coating air holes in microstructure optical fibers has been successfully demonstrated in experiments. The vapor-deposition method [33], high-pressure microfluidic chemical-deposition technology [32], and the magnetron-sputtering method [34] are widely used for metal coating. The vapor-deposition method is a common technology in metal-film coating. The high-pressure microfluidic chemical-deposition technology can achieve uniform, dense, and annular sediments in the PCF air holes. The magnetron-sputtering method is a type of Physical Vapor Deposition (PVD). It has the advantages of simple equipment, easy control, uniform coating, and strong adhesion. The air hole of the sensing Channel 2 can be coated by the high-pressure microfluidic chemical-deposition technology. The D-type plane of the sensing Channel 1 can be coated by the magnetron-sputtering method. The fabrication process of the PCF-SPR is shown in Figure 2.

**Figure 3:**
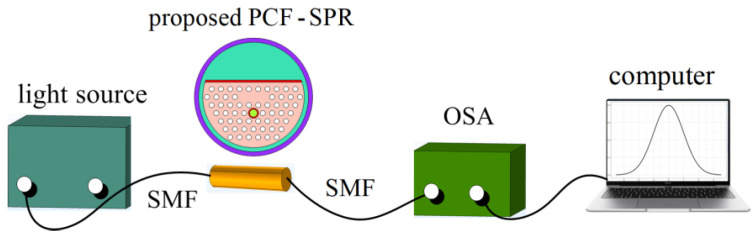
Schematic of the proposed PCF-SPR RI sensor setup.

Figure 3 shows the experimental setup for the practical realization of the PCF-SPR RI sensing. The light source produces broadband continuous spectrum and launches light into the single mode fiber (SMF). The SMF transmits the light to the PCF-SPR. In the process of interaction between light and analyte, part of the energy of the light signal is absorbed. The loss information is transmitted to an optical spectrum analyzer (OSA) through another SMF. Finally, the results of calculation and analysis are obtained by a computer.

**Figure 4:**
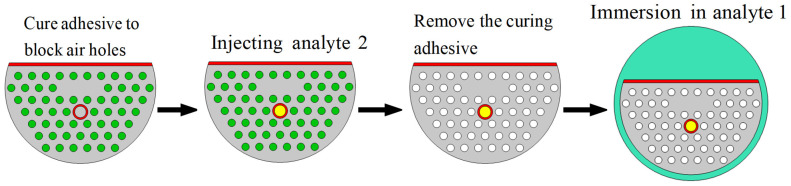
Flow chart of double-sample detection. The white and green circles represent air holes and cured adhesives, respectively. The gold layer is marked in red. Analyte 1 and Analyte 2 are labeled blue and yellow, respectively.

Figure 4 describes the specific steps of dual-sample detection. Firstly, the air holes that do not need to be filled should be blocked with curing adhesive, and then the analyte 2 is filled into Channel 2 by the capillary-absorption phenomenon. Next, the section of the blocked PCF should be cut off to remove the curing adhesive. Finally, connect the PCF-SPR to the experimental setup illustrated in Figure 3 and submerge the D-type PCF in Analyte 1 for detection. In order to avoid polluting the analyte and ensure the cleanness of the sensing area, the sensor should be repeatedly cleaned with alcohol before and after each test.

In the simulation, we use FEM to conduct structural modeling and mode characteristic analysis through the analysis software COMSOL Multiphysics 5.5. In order to reduce the impact of electromagnetic wave reflection at the computational interface on the computational results, a perfectly matched layer (PML) is added to the outer periphery of the cladding to absorb incident electromagnetic waves. As shown in Figure 1, PML is a cylindrical surface with a thickness of 1000 nm, and the inner radius and outer radius are 12,000 nm and 13,000 nm, respectively. The RI of the material constituting the PML is set to 1.5. At the same time, the scattering boundary condition (SBC) is used to deal with the problem of energy reflection at the boundary. In this paper, free triangular meshes are used to discretize the whole solution domain due to the versatility and ease of control. The whole mesh contains 20,253 domain elements and 1946 boundary elements, with 251 vertex elements. The maximum cell size is 2600 nm, and the minimum cell size is 52 nm.

The background material of the PCF is fused quartz, and its material dispersion model is expressed by the Sellmeier equation [35], as follows:

(1) $$n^{2}\left(\lambda \right)=1+\frac{B_{1}\lambda^{2}}{\lambda^{2}-C_{1}}+\frac{B_{2}\lambda^{2}}{\lambda^{2}-C_{2}}+\frac{B_{3}\lambda^{2}}{\lambda^{2}-C_{3}}$$

where *n* is the RI of fused quartz, *λ* is the working wavelength (in m), and *B_i_* and *C_i_* are the Sellmeier coefficients for *i* = 1, 2, 3. The values of Sellmeier Coefficients *B*_1_, *B*_2_, *B*_3_, *C*_1_, *C*_2_, and *C_3_* are 0.6961663, 0.4079426, 0.8974794, 4.6791482 × 10^−15^ m^2^, 1.35120631 × 10^−14^ m^2^, and 9.79340025× 10^−11^ m^2^, respectively. The dielectric constant of gold can be obtained through the Drude–Lorentz model [36,37]. The RI of the air in the air hole is 1. The confinement loss of the mode can be calculated by the following formula [38]:

(2) α=8.686×2πλ×Im(neff)×107dB/cm

where λ is the wavelength of the incident light, the unit is nm, and Im (*n_eff_*) is the imaginary part of the effective RI.

## 3. Simulation Results and Discussion

### 3.1. Mode Analysis of Photonic Crystal Fiber

**Figure 5:**
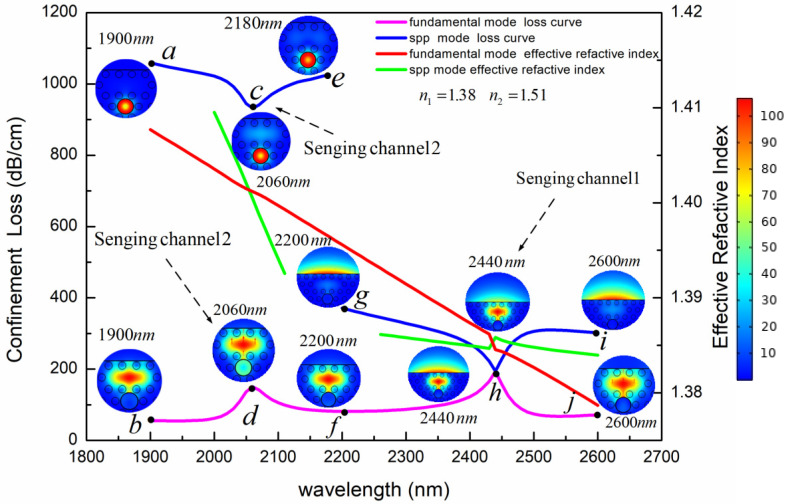
The confinement loss and dispersion relationship of the fundamental mode and SPP mode in the two sensing channels at *n*_1_ = 1.38 and *n*_2_ = 1.51. Insets are electric field distributions of various wavelengths. The color of the insets represents the strength of electric field, and its unit is v/m.

In order to analyze the mode characteristics of the PCF-SPR sensor, we calculated the confinement loss and dispersion relations of the fundamental mode and surface plasmon polaritons (SPP) mode for two sensing channels. Since the sensing performance of the y polarization mode is better than that of x polarization mode, we only discuss the y polarization mode in this paper. The calculation results are shown in Figure 5 with the parameters d_1_ = 1080 nm, d_2_ = 2400 nm, Ʌ = 2000 nm, h = 2400 nm, c = 1000 nm, t_1_ = 50 nm, t_2_ = 60 nm, n_1_ = 1.38, and n_2_ = 1.51. The illustrations depict the electric field distribution corresponding to different wavelengths. As shown in Figure 5, the short wavelength segment on the left shows the mode characteristics of Channel 2, while the long wavelength segment on the right shows the mode characteristics of Channel 1. For Channel 2, the dispersion curves of the two modes have an intersection, which is called the phase-matching point. According to the illustrations of electric field distribution at Points a–f in the band far away from the phase-matching point, both the fundamental mode and the SPP mode are well confined to their respective regions. When approaching the phase-matching point, a small amount of energy is transferred between the two modes. The largest energy transfer occurs at the phase-matching point. This is called incomplete coupling. The coupling characteristics of the two modes in Channel 1 are different from those in Channel 2. At the h point with the wavelength of 2440 nm, the confinement losses of the fundamental mode and the SPP mode are equal, and the effective RI difference is the smallest. The phase-matching condition and loss-matching condition are satisfied at the same time. The energy is completely transferred between the two modes. At the h point, there is nearly the same electric field distribution for the fundamental mode and the SPP mode. This is called complete coupling.

**Figure 6:**
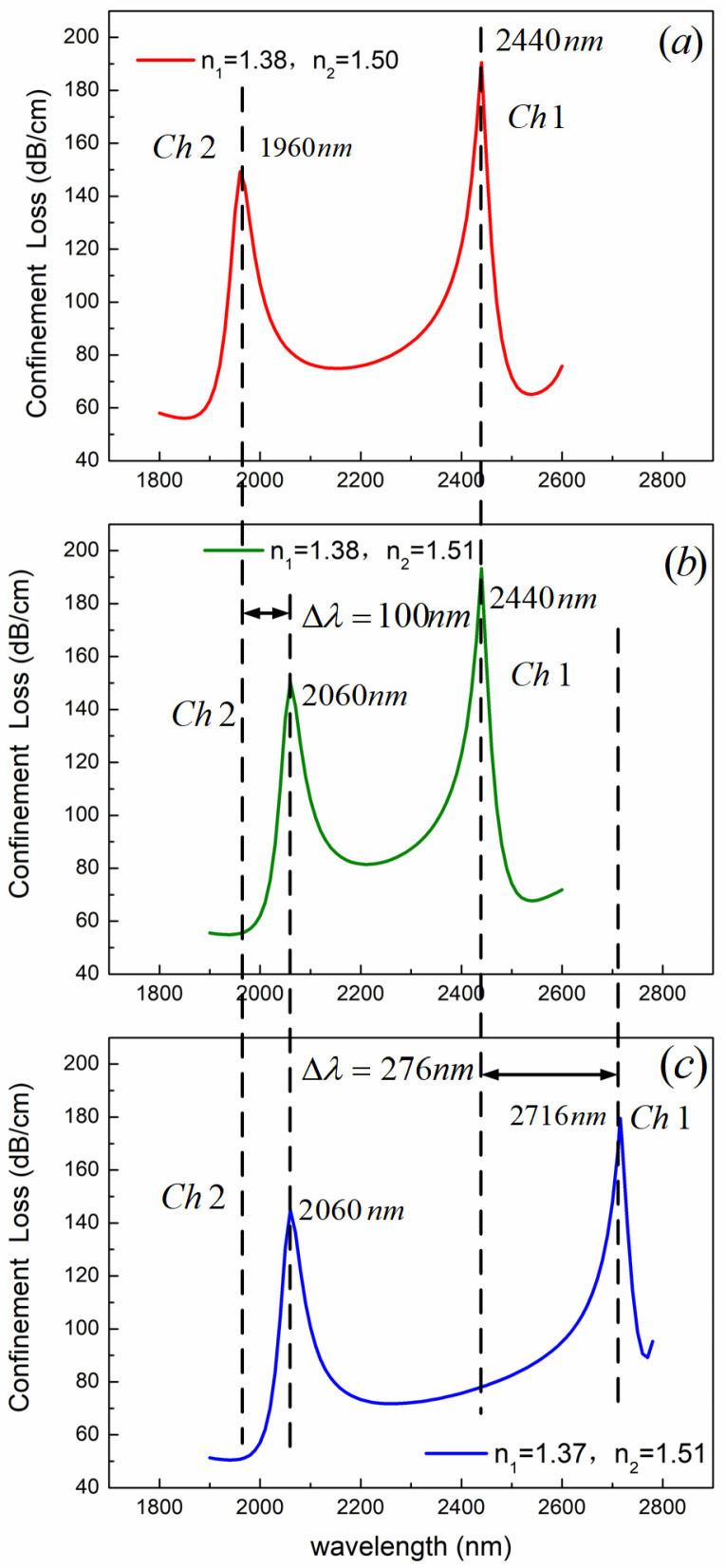
Resonance peak versus RI of the two sensing channels for (**a**) *n*_1_ = 1.38, *n*_2_ = 1.50, (**b**) *n*_1_ = 1.38, *n*_2_ = 1.51, and (**c**) *n*_1_ = 1.37, *n*_2_ = 1.51.

The control-variable method is used to study the sensing performance of the two channels. Other structural parameters remain unchanged, as above. The shift of the resonance peak is shown in Figure 6 by changing the RI of analytes in Channel 1 and Channel 2, respectively. As shown in Figure 6, the left side is the resonance peak of Channel 2, and the right side is the resonance peak of Channel 1. When the analyte RI in Channel 1 remains unchanged and the analyte RI in Channel 2 changes from 1.50 to 1.51, the resonant wavelength of Channel 1 remains unchanged at 2440 nm, while the resonant wavelength of Channel 2 shifts by 100 nm. This indicates that the change in analyte RI of Channel 2 has no effect on the resonant wavelength of Channel 1. When the RI of the analyte in Channel 2 remains unchanged, and the RI of the analyte in Channel 1 changes from 1.37 to 1.38, the resonant wavelength of Channel 1 shifts by 276 nm, while the resonant wavelength of Channel 2 remains unchanged at 2060 nm. This indicates that the change in RI of the analyte in Channel 1 has no effect on the resonant wavelength of Channel 2. The above results show that the two sensing channels will not affect each other. The two sensing channels are independent of each other and can simultaneously measure two analytes with different RIs.

### 3.2. Effect of Structural Parameters on Sensing Performance

The optimization of structural parameters is crucial for the stability and effective sensing response of sensors. Typically, confinement loss is one of the best parameters for considering the performance of PCF-SPR sensors, so confinement loss is selected as the sensing parameter in this study to optimize structural parameters and evaluate sensing performance. The following discusses the influence of structural parameters on sensor performance. By optimizing parameters, the sensing performance can be optimized.

**Figure 7:**
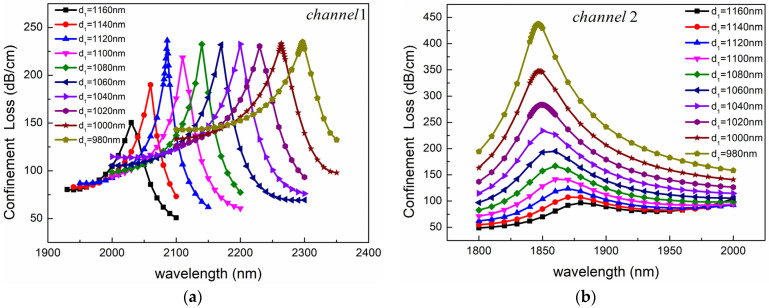
Confinement loss spectra for different air-hole diameters of (**a**) Channel 1 and (**b**) Channel 2. The other structural parameters are *n*_1_ = 1.39, *n*_2_ = 1.49, *d*_2_ = 2400 nm, *c* = 1000 nm, *Ʌ* = 2000 nm, *h* = 2400 nm, *t*_1_ = 50 nm, and *t*_2_ = 60 nm.

Firstly, the influence of air-hole diameter on sensing performance was analyzed. With other parameters fixed, the confinement loss of the fundamental mode was calculated when the air-hole diameter increased from 980 nm to 1160 nm. The results are shown in Figure 7. As can be seen from Figure 7, the influence of the air-hole diameter on the sensing performance of the two channels is completely different. As shown in Figure 7a, with the increase of d_1_, the resonance peak of Channel 1 shows a blue shift, and the spectral width of the peak first decreases and then increases. When d_1_ is less than 1120 nm, the peak value is almost unchanged, and when d_1_ is greater than 1120 nm, the peak value decreases significantly. This is because, with the increase of the diameter of the air hole, the effective RI of the mode changes, and the phase-matching point moves to the short wavelength direction. The situation of Channel 2 is different from that of Channel 1, as shown in Figure 7b. As d_1_ decreases, the position of the resonance peak remains almost unchanged, while the peak value gradually increases. This is because the decrease in air holes promotes the generation of SPR effects in the optical fiber, resulting in an increase in the mode field around the gold nanolayer and an increase in confinement loss.

**Figure 8:**
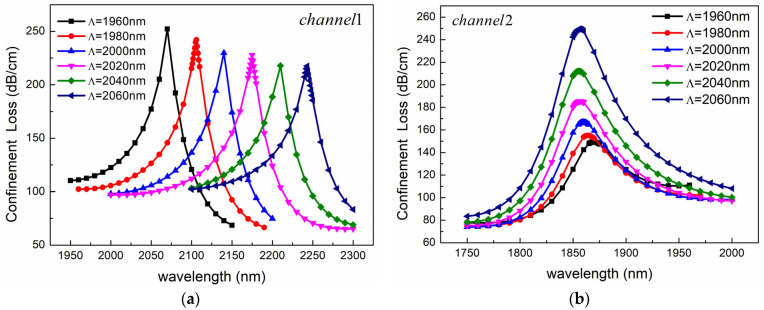
Confinement loss spectra for different hole spacings of (**a**) Channel 1 and (**b**) Channel 2. The other structural parameters are *n*_1_ = 1.39, *n*_2_ = 1.49, *d*_1_ = 1080 nm, *d*_2_ = 2400 nm, *c* = 1000 nm, *h* = 2400 nm, *t*_1_ = 50 nm, and *t*_2_ = 60 nm.

The effect of hole spacing on sensing performance is also considerable. We calculated the change of confinement loss when the hole spacing Λ increased from 1960 nm to 2060 nm, and other parameters remained unchanged. As shown in Figure 8, with the increase of hole spacing, the fiber core loss of Channel 1 decreases, while that of Channel 2 increases, indicating that the change of hole spacing has the opposite effect on the peak value of the two channels. In addition, with the increase of hole spacing, the resonant wavelength in Channel 1 is red-shifted, while the position of the resonant wavelength in Channel 2 is almost unchanged. Therefore, the change of hole spacing changes the phase-matching point in Channel 1 but has no effect on the phase-matching point in Channel 2.

**Figure 9:**
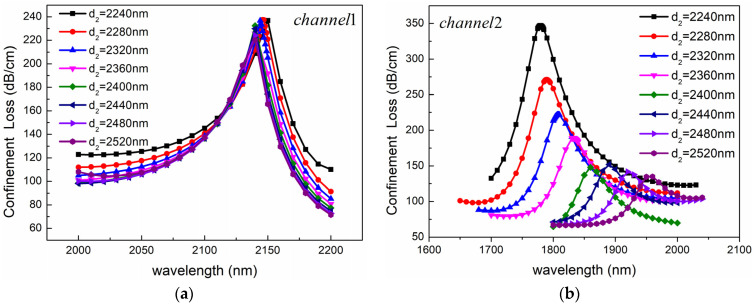
Confinement loss spectra for various Channel 2 diameters of (**a**) Channel 1 and (**b**) Channel 2. The other structural parameters are *n*_1_ = 1.39, *n*_2_ = 1.49, *d*_1_ = 1080 nm, *Ʌ* = 2000 nm, *c* = 1000 nm, *h* = 2400 nm, *t*_1_ = 50 nm, and *t*_2_ = 60 nm.

Figure 9 depicts the effect of sensing Channel 2 diameter on sensing performance. As shown in Figure 9a, the change of the diameter of the sensing channel 2 has little effect on the resonant peak of Channel 1. Only with the increase of the diameter of Sensing Channel 2, the spectral width of the resonant peak gradually decreases. It can be seen from Figure 9b that the change in the diameter of Sensing Channel 2 has a more obvious impact on Channel 2. With the increase of the diameter of Channel 2, the resonance peak decreases significantly, and the resonance wavelength moves to the long wavelength direction. This indicates that the change of Channel 2’s diameter causes the change of effective RI of the modes. Moreover, the larger diameter of Channel 2 makes the core mold well confined in the core area, and the energy leakage is reduced.

**Figure 10:**
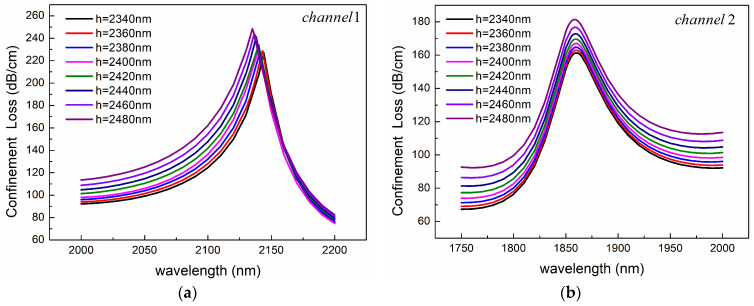
Confinement loss spectra for different polishing depths of (**a**) Channel 1 and (**b**) Channel 2. The other structural parameters are *n*_1_ = 1.39, *n*_2_ = 1.49, *d*_1_ = 1080 nm, *d*_2_ = 2400 nm, *Ʌ* = 2000 nm, *c* = 1000 nm, *t*_1_ = 50 nm, and *t*_2_ = 60 nm.

The polishing depth determines the distance between the core and the plasma material, so it affects the coupling efficiency between the core mode and the SPP mode. As shown in Figure 10a, with the increase of polishing depth, the resonance peak value of Channel 1 slightly increases, the resonance wavelength shifts slightly to the blue, and the waveform becomes wider. For Channel 2, the increase of polishing depth only changes the height of the resonance peak but does not change the position of the resonance wavelength, as shown in Figure 10b.

**Figure 11:**
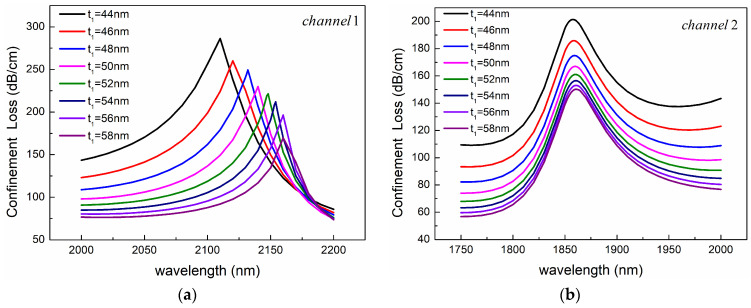
Confinement loss spectra for different gold-layer thickness *t*_1_ of (**a**) Channel 1 and (**b**) Channel 2. The other structural parameters are *n*_1_ = 1.39, *n*_2_ = 1.49, *d*_1_ = 1080 nm, *d*_2_ = 2400 nm, *Ʌ* = 2000 nm, *c* = 1000 nm, *h* = 2400 nm, and *t*_2_ = 60 nm.

Figure 11a shows the effect of the gold-layer thickness t_1_ on the loss spectrum of channel 1. With the increase of gold-film thickness t_1_, the loss spectrum shows a significant redshift. The reason is that the increase in the thickness of the gold layer reduces the effective RI of the SPP mode, while the effective RI of the core mode is almost unchanged so that the phase-matching point moves towards the long-wave direction. In addition, Figure 11a,b both show that the resonance peak decreases with the increase of the thickness of the gold layer. As we all know, when the thickness of the gold film becomes larger, the evanescent wave excited by the evanescent field is more difficult to pass through the metal layer, resulting in the reduction of coupling efficiency and resonance strength.

**Figure 12:**
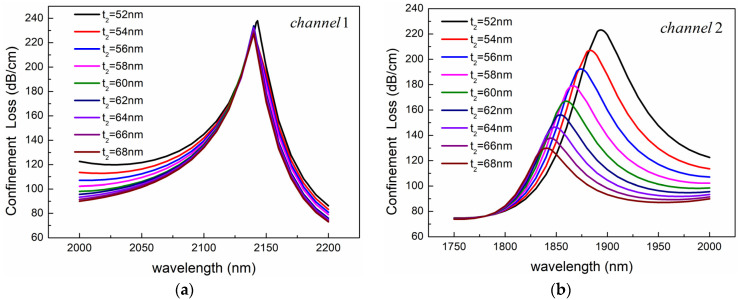
Confinement loss spectra for different gold-layer thickness *t*_2_ of (**a**) Channel 1 and (**b**) Channel 2. The other structural parameters are *n*_1_ = 1.39, *n*_2_ = 1.49, *d*_1_ = 1080 nm, *d*_2_ = 2400 nm, *Ʌ* = 2000 nm, *c* = 1000 nm, *h* = 2400 nm, and *t*_1_ = 50 nm.

The influence of the gold-layer thickness of Channel 2 on the sensing performance of the two channels is shown in Figure 12. As shown in Figure 12a, the thickness of the gold layer has little effect on the resonance peak of Channel 1, only changing the width of the peak. Compared with Channel 1, the effect of gold-layer thickness on Channel 2 is more significant as shown in Figure 12b. In Channel 2, the dispersion curve of the SPP mode is steeper than that of the fundamental mode. Therefore, as the thickness of the gold layer increases, the RI of the SPP mode gradually decreases, while the RI of the fundamental mode remains almost unchanged, resulting in a blue shift in the resonant wavelength. At the same time, as the thickness of the gold layer increases, the resonance peak gradually decreases due to the increased difficulty of evanescent waves passing through the gold layer.

**Figure 13:**
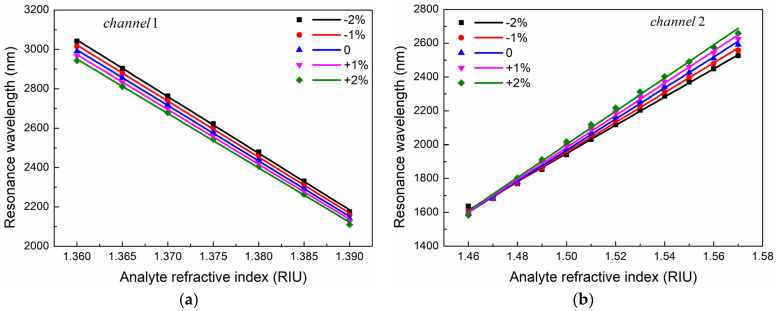
Effect of fabrication deviation on (**a**) Channel 1 and (**b**) Channel 2.

The above calculation and analysis show that the influence of fiber-structure parameters on the loss curves of the two channels is different, even opposite. This can be observed from the impact of changes in parameters on wavelength sensitivity in Figure 13. Furthermore, simulation results vary depending on the specific parameter combinations. We determine the RI detection range of the two channels according to the characteristics of the loss curves. Considering the impact of structural parameters on the FWHM of the loss curve, the RI detection range of the two channels, and the sensitivity, we choose *d_1_* = 1080 nm, *d_2_* = 2400 nm, *Ʌ* = 2000 nm, *h* = 2400 nm, *t_1_* = 50 nm, *t_2_* = 56 nm, and *c* = 1000 nm as the optimal parameters for the simulation.

In the process of drawing optical fibers, there will be unavoidable variations of 1% to 2% in the diameter of the air holes (*d_1_*), Channel 2 diameter (*d_2_*), polishing depth (*h*), and thickness of the gold film (*t_1_*, *t_2_*) [39]. These variations must be considered. Figure 13 shows the impact of these deviations on wavelength sensitivity based on optimized structural parameters. It has been observed that within the manufacturing deviation range of ±2%, the resonant wavelength of the sensor drifts slightly due to the deviation, but the overall trend changes minimally. This means that the impact on the wavelength sensitivity is small. It is evident that the sensor exhibits stable sensing performances.

### 3.3. Performance Analysis

The basic principle of the PCF-SPR RI sensor is based on the interaction between the core mode and the SPP mode. When the real parts of effective RI of the core mode and the SPP mode are equal, the phase-matching condition is met and strong coupling between the two modes occurs. The energy of the core mode is transferred to the SPP mode, forming the SPR effect. The loss curve of the core mode shows a peak. This resonant absorption peak is very sensitive to changes in the RI of the analyte. Therefore, it can be used to measure the RI of analytes.

**Figure 14:**
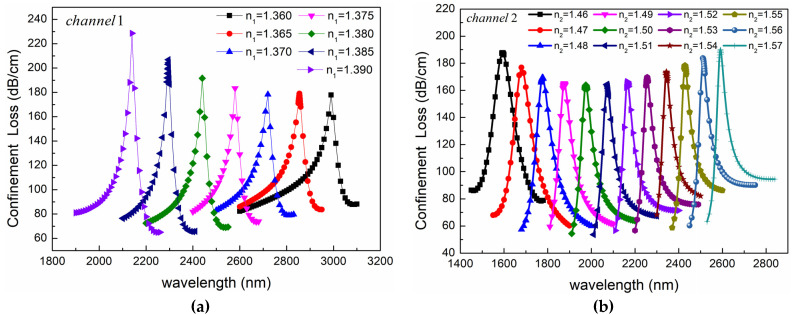
Confinement losses for different analytes in the RI ranges of (**a**) 1.36–1.39 for Channel 1 and (**b**) 1.46–1.57 for Channel 2.

First, we calculated the sensing characteristics of Channel 1. In the simulation, it was considered that Channel 2 was not filled and could be regarded as a large pore. We calculated the relationship between the confinement loss and wavelength by changing the RI of the analyte in Channel 1 with the structural parameters of the fiber unchanged. The results show that the loss peak has good characteristics when the RI of the analyte in Channel 1 is in the range of 1.36–1.39. The simulation results are shown in Figure 14a. Because the dispersion curve of the fundamental mode is steeper than that of the SPP mode in channel 1, and as the RI of the analyte increases, the RI of the SPP mode increases. While the RI of the fundamental mode remains almost unchanged, the phase-matching point will shift towards shorter wavelengths as the RI of the analyte increases, resulting in a blue shift, as shown in Figure 14a. In addition, with the increase of the RI of the analyte, the loss peak increases significantly, indicating that the higher RI of the analyte enhances the resonance between the fundamental mode and the SPP mode.

Next, we studied the sensing characteristics of Channel 2. At this time, we assumed that the analyte in Channel 1 was air, and other structural parameters remained unchanged. We calculated the variation of the resonance peak with the RI of the analyte in Channel 2, as shown in Figure 14b. The simulation results show that the resonance peak has excellent characteristics when the RI of the analyte in Channel 2 is in the range of 1.46–1.57. Unlike Channel 1, in Channel 2, the dispersion curve of the fundamental mode is flatter than that of the SPP mode. When the RI of the analyte in Channel 2 increases, the RI of the SPP mode increases, while the RI of the fundamental mode is basically unchanged. Therefore, the intersection of the dispersion curves of the two modes will move towards the long wave direction. The resonance wavelength is red-shifted. In addition, as the RI of the analyte increases, the loss peak first decreases and then increases.

According to the above analysis, when the analyte RI in one of the two channels is fixed to 1, the other can be used as an independent detection channel and can be used alone. When Channel 1 is used independently, it can be used as a low-RI detection channel with an RI detection range of 1.36–1.39 in the wave band of 2100–3000 nm. Similarly, when Channel 2 is used independently, it can be used as a high-RI detection channel with an RI detection range of 1.46–1.57 in the wave band of 1500–2600 nm. In addition, the two sensing channels can also detect two analytes with different RIs at the same time. Since the resonance peaks of the two channels overlap within the detection range, the detection range will be reduced when detecting simultaneously compared with independent detection. For example, if the detection range of Channel 1 remains 1.36–1.39, the detection range of Channel 2 will be reduced to 1.46–1.51.

**Figure 15:**
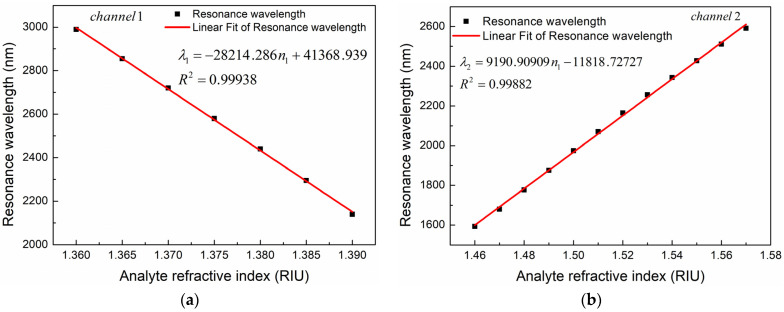
Variation of resonance wavelength with analyte RI as the RI is changed from (**a**) 1.36 to 1.39 in Channel 1 and (**b**) 1.46 to 1.57 in Channel 2.

Wavelength sensitivity is defined as the rate of change in resonant wavelength relative to RI and is an important parameter for evaluating sensor performance. Wavelength sensitivity can be calculated by the following formula [11].

(3) $$S_{\lambda }\left(\lambda \right)=\partial \lambda /\partial n\left[nm/RIU\right]$$

where ∂λ is the change of resonance wavelength caused by the change of analyte RI, and ∂n is the change of analyte RI. Obviously, the slope of the fitting curve obtained by fitting the scatter diagram of resonance wavelength varying with RI is the wavelength sensitivity of the sensor. We fitted the relationship between resonance wavelength and analyte RI of the two channels, and the fitting results are shown in Figure 15. It can be seen that there is a good linear relationship between the resonant wavelength and the RI of the analyte in both channels. The linear fitting equation and the adjusted R-square (ARS) values of the two channels are as follows:

(4) $$\lambda_{1}=-28214.286n_{1}+41368.939,\;1.36\leq n_{1}\leq 1.39,\;ARS_{1}=0.99938$$

(5) $$\lambda_{2}=9190.90909n_{2}-11818.72727,\;1.46\leq n_{2}\leq 1.57,\;ARS_{2}=0.99882$$

where $\lambda_{1}$ and $\lambda_{2}$, respectively, represent the resonance wavelength of Channel 1 and Channel 2, and $n_{1}$ and $n_{2}$ are the RIs of the analytes in Channel 1 and Channel 2, respectively. Obviously, the slope of the linear fitting curve represents the average wavelength sensitivity of the sensor. According to the fitting results in Figure 15a, when n_1_ changes from 1.36 to 1.39, the average wavelength sensitivity in Channel 1 is 28,214.286 nm/RIU. The maximum wavelength sensitivity of Channel 1 is up to 30,000 nm/RIU, which appears near $n_{1}$ = 1.38. As shown in Figure 15b, when the RI of the analyte in Channel 2 increases from 1.46 to 1.57, the average wavelength sensitivity of Channel 2 is 9190.90909 nm/RIU. The maximum wavelength sensitivity of Channel 2 can reach 9900 nm/RIU when $n_{2}$ = 1.49.

The RI resolution of the sensor is also an important parameter to evaluate the performance of the sensor, which determines the ability of the sensor to recognize small alterations in analyte RI. The RI resolution of the sensor can be calculated by the following formula [11]:

(6) $$R=\partial n\times \partial \lambda_{\min}/\partial \lambda \left[RIU\right]$$

where $\partial \lambda_{min}$ is the wavelength resolution of the instrument used. If a spectrometer with the optimal wavelength resolution of 0.1 nm is used (such as Yokogawa AQ6376 fiber spectrometer), the RI resolution of the sensor can reach 3.54 × 10^−6^ RIU in Channel 1 and 10.88 × 10^−6^ RIU in Channel 2, respectively.

The *FOM* and *FWHM* are important parameters for measuring the performance of sensors. FWHM is the wavelength band corresponding to 1/2 of the height of the loss peak. The smaller the *FWHM*, the smaller the transmission loss of the sensor in the non-resonant wavelength range. *FOM* is defined as the ratio of wavelength sensitivity to *FWHM*. Generally, in order to ensure that the PCF-SPR sensor has a higher *FOM*, it is necessary to improve the wavelength sensitivity, and the *FWHM* value should be as low as possible. *FOM* can be calculated using the following equation [11]:

(7) $$FOM=\frac{S_{\lambda }}{FWHM}$$

where $S_{\lambda }$ is the wavelength sensitivity of the sensor.

**Figure 16:**
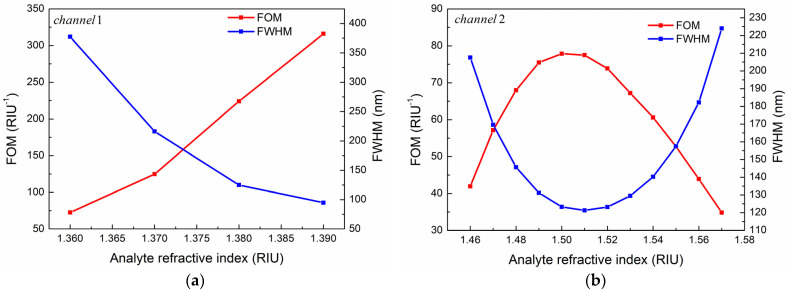
Relationship between *FOM*, *FWHM*, and RI of analytes in (**a**) Channel 1 and (**b**) Channel 2.

In this paper, the *FWHM* and *FOM* of the two sensing channels are calculated, respectively, as shown in Figure 16. It can be seen from Figure 16a that in Channel 1, *FOM* increases with the increase of analyte RI, while FWHM has the opposite trend. When n_1_ = 1.39, *FOM* reaches the maximum value of 315.87. In Channel 2, as the RI of the analyte increases, the *FOM* increases first and then decreases, reaching the maximum value of 77.87 at n_2_ = 1.50. *FWHM* has a trend opposite to *FOM* as shown in Figure 16b.

In addition to wavelength sensitivity, RI resolution, *FWHM*, and *FOM*, *SNR* is also an important indicator for evaluating the performance of the PCF-SPR sensor. The higher the *SNR*, the higher the accuracy of the sensor measurement. The calculation formula of the *SNR* is as follows [40]:

(8) $$SNR=\frac{\Delta \lambda_{R}}{FWHM}$$

where $\Delta \lambda_{R}$ is the difference between resonance wavelengths corresponding to two adjacent RIs.

**Figure 17:**
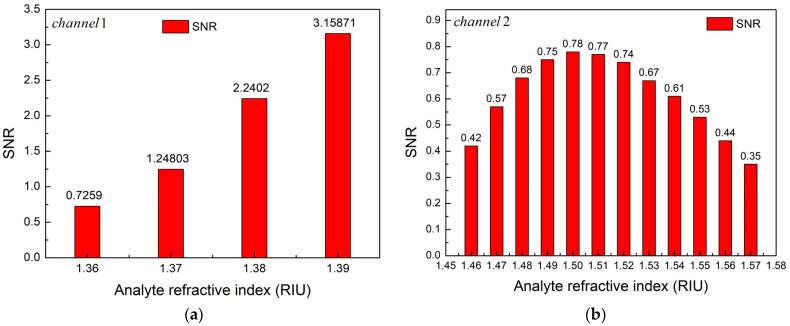
Relationship between *SNR* and analyte RI in (**a**) Channel 1 and (**b**) Channel 2.

Figure 17 depicts the relationship between *SNR* and RI of the analyte in two channels. According to Figure 17a, in Channel 1, the *SNR* gradually increases with the increase of the analyte RI. When $n_{1}$ = 1.39, the SNR reaches the maximum value of 3.15871. For Channel 2, as the RI of the analyte increases, the *SNR* first increases and then decreases. At $n_{2}$ = 1.50, the *SNR* reaches the maximum value of 0.78, as shown in Figure 17b. Obviously, the *SNR* and *FOM* have the same trend of change.

Table 1 shows the comparison between our proposed dual-channel PCF-SPR RI sensor and the previously reported sensors in the literature [41,42,43,44,45,46]. Obviously, the sensor proposed in this paper has excellent performance in the RI detection range, operation wavelength, average wavelength sensitivity, and RI resolution, as shown in Table 1. The vast majority of previously reported sensors have the same RI measurement range for both channels, and the range is relatively narrow. The sensor proposed in this paper has two channels with different RI measurement ranges: 1.36–1.39 and 1.46–1.57. This setup allows for an independent detection of analytes with varying RI levels and simultaneous detection of two analytes with different RI. The large RI measurement range increases the application prospect of the sensor. The previously reported PCF-SPR sensor can only work in visible light and near-infrared bands. It rarely works in the mid-infrared band, and the working wavelength range is narrow. The sensor proposed in this paper can operate in the ultra-wide wavelength range of 1500–3000 nm, which means that the sensor can work simultaneously in the near-infrared and mid-infrared regions. Compared with the visible light band, the near-infrared and mid-infrared bands have unique advantages [47,48] as the working wavelengths of PCF-SPR sensors. Sensors operating in the near-infrared and mid-infrared bands can avoid light damage and phototoxicity to biological materials [49,50]. In addition, the penetration depth of evanescent waves is directly proportional to the working wavelength. Compared with visible light, the penetration depth of evanescent waves in the near-infrared and mid-infrared bands is deeper, which can improve the detection sensitivity of sensors for biological macromolecular targets [51,52]. Therefore, the sensor proposed in this paper has significant advantages and broad application prospects.

## 4. Conclusions

In this paper, a novel dual-channel PCF-SPR RI sensor is proposed. The structural parameters and sensing characteristics are studied by full-vector FEM. The calculation results show that the dual-channel sensor can achieve high-sensitivity synchronous detection of analytes with different RIs in the ultra-long band from near-infrared to mid-infrared. When Channel 1 is used independently, it can be used as a low-RI detection channel, with an RI detection range of 1.36–1.39 in the wave band of 2100–3000 nm. Similarly, when Channel 2 is used independently, it can be used as a high-RI detection channel, with an RI detection range of 1.46–1.57 in the wave band of 1500–2600 nm. In addition, the two sensing channels can also detect two analytes with different RI at the same time. The average wavelength sensitivity of the sensor is 28,214.286 nm/RIU in Channel 1 and 9190.9 nm/RIU in Channel 2, respectively. The maximum wavelength sensitivity is up to 30,000 nm/RIU in Channel 1 and 9900 nm/RIU in Channel 2. The RI resolutions of the two channels are 3.54 × 10^−6^ RIU and 10.88 × 10^−6^ RIU, respectively. In conclusion, the proposed sensor achieves an ultra-wide detection range for RI and high sensitivity in the ultra-long band from near-infrared to mid-infrared. The sensor has considerable sensing performance and broad application prospects, which provides a theoretical reference for the design of dual-channel RI sensors.

## Figures and Tables

**Table 1:** Performance comparison of the proposed sensor with the previously reported sensors.

References	Channel	RI Range (RIU)	Wavelength Range (nm)	$S_{\lambda }$ (nm/RIU)	RI Resolution (RIU)	FOM (RIU^−1^)	Research Types
[41]	Ch 1	1.34–1.36	528–614	4280	2.34 × 10^−5^	N/A	Numerical
	Ch 2	1.34–1.36	658–738	3940	2.54 × 10^−5^	N/A	
[42]	Ch 1	1.33–1.366	400–800	1892	4 × 10^−5^	N/A	Numerical
	Ch 2	1.33–1.366	400–800	2337	3.2 × 10^−5^	N/A	
[43]	Ch 1	1.31–1.41	1350–1600	6000	5 × 10^−5^	125	Numerical
	Ch 2	1.31–1.41	1450–1650	6000	5 × 10^−5^	68.96	
[44]	Ch 1	1.33–1.40	530–970	12,000	8.33 × 10^−6^	200	Numerical
	Ch 2	1.33–1.39	530–970	10,000	1.0 × 10^−5^	145	
[45]	Ch 1	1.33–1.39	600–1500	7540	1.3 × 10^−5^	522	Numerical
	Ch 2	1.33–1.39	600–1500	7540	1.3 × 10^−5^	280	
[46]	Ch 1	1.3253–1.3726	700–850	2496	N/A	N/A	Experimental
	Ch 2	1.5255–1.5781	560–700	1951			
This work	Ch 1	1.36–1.39	2100–3000	28,214.286	3.54 × 10^−6^	315.87	Numerical
	Ch 2	1.46–1.57	1500–2600	9190.9	10.88 × 10^−6^	77.87	

## Data Availability

Data are contained within the article.
